# RNF180 weakened the lipid droplet formation and subsequent chemoresistance by destabilizing ACC1 and ACLY in esophageal cancer

**DOI:** 10.3389/fphar.2025.1525431

**Published:** 2025-04-22

**Authors:** Ning Li, Dao-Fu Shen, Nan-Chang Yin, Zheng-Guo Cui, Hua-Chuan Zheng

**Affiliations:** ^1^ Center of Translational Medicine and Cancer Center, The First Affiliated Hospital of Jinzhou Medical University, Jinzhou, China; ^2^ Department of Clinical Laboratory, Chifeng Municipal Hospital, Chifeng, China; ^3^ Department of Thoracic Surgery, The First Affiliated Hospital of Jinzhou Medical University, Jinzhou, China; ^4^ Department of Environmental Health, University of Fukui School of Medical Sciences, Fukui, Japan

**Keywords:** RNF180, drug resistance, lipid droplet formation, aggressiveness, target therapy, esophageal cancer

## Abstract

**Objective:**

RNF180 (Ring finger protein 180) is an E3 ubiquitin-protein ligase that promotes polyubiquitination and proteasomal degradation. The study aimed to clarify the clinicopathological significances, signal pathways and molecular mechanisms of RNF180 expression in esophageal cancer.

**Methods:**

We analyzed the clinicopathological significances and signal pathways of RNF180 expression in esophageal cancer (EC) through bioinformatics and pathological analysis. We also clarified its effects on aggressiveness and related molecular mechanisms *in vitro*.

**Results:**

RNF180 mRNA expression was lower in EC than in normal tissues (p < 0.05), opposite for its methylation (p < 0.05). *RNF180* mRNA expression was negatively correlated with its promoter methylation, but positively with high histological grading, N stage, and poor prognosis of EC (p < 0.05). RNF180 protein expression was positively associated with T stage, N stage, and TNM stage, but negatively with unfavorable overall survival of EC as an independent factor (p < 0.05). The differential genes of *RNF180* can be categorized into olfactory transduction, focal adhesion, vascular smooth muscle contraction, calcium signal pathway, cell adhesion molecules, muscle contraction, ECM receptor interaction, and collagen degradation (p < 0.05). *RNF180*-related genes can be categorized into gastric acid and insulin section, muscle and cardiomyopathy, glycoprotein binding, collagen and extracellular matrix, fat digestion and diabetes, PPAR signal pathway and peptidase activity. RNF180 overexpression reduced proliferation, migration, invasion and epithelial-mesenchymal transition, and induce mitochondrial apoptosis, and Caspase-1-dependent pyroptosis of EC cells (p < 0.05). RNF180 might induce chemosensitivity by weakening ACC1- and ACLY-mediated lipogenesis *via* the ubiquitination and proteasomal degradation of ACC1 and ACLY, and lipid droplet assembly.

**Conclusion:**

*RNF180* might be considered as a biological marker for aggressive behaviors and poor prognosis in EC and as a molecular target of gene therapy.

## 1 Introduction

Among cancer deaths worldwide, esophageal cancer (EC) ranks sixth (Musa et al., 2021). Various genetic mutations drive the development of squamous cell carcinoma (SCC) and adenocarcinoma (AD), the two most common types of EC (Zhao et al., 2021). While EC can be treated in a variety of ways, including surgery, chemotherapy, or radiotherapy, the general outcome remains poor (Musa et al., 2021; Mwachiro et al., 2021; Zhao et al., 2021). Hence, it may be of therapeutic benefit to find out the biomarkers and molecular targets of EC.

*RNF180* (Ring finger protein 180) is located in human chromosome 5q12.3, encoded E3 ubiquitin ligase (Ogawa et al., 2008). In vertebrates, RNF180 contains a RING finger domain, a basic coiled-coil domain, a novel conserved domain (DSPRC), and a transmembrane hydrophobic region (Ogawa et al., 2008). N-terminal epitope-tagged-Rines are integral membrane proteins, most of which is distributed to the cytoplasm of the endoplasmic reticulum (Ogawa et al., 2008). Wu et al. (2020) found that RNF180 suppressed STAT3 phosphorylation *via* the ubiquitination and proteasomal degradation of RhoC in gastric cancer cells. Wei et al. (2021) reported that WISP1 was ubiquitinated by RNF180, which ultimately suppressed tumor growth in colorectal cancer cells. Cao et al. (2021) demonstrated that PLK2 was interacted with and ubiquitinated by RNF180. RNF180 upregulation induced apoptosis in glioma cells, which was significantly inhibited by PLK2 overexpression (Cao et al., 2021). Sun L. et al. (2021) showed that DNA methyltransferase 3α (DNMT3A) was ubiquitinated by RNF180 and then degraded in proteasome of gastric cancer cells.

Reportedly, *RNF180* is expressed in adult mice’s brains, kidneys, testes, uteri, developing lenses, and brain (Ogawa et al., 2008). Its hypoexpression was observed in gastric and colorectal cancers due to its promoter of CpG methylation (Wei et al., 2021; Cheung et al., 2012). Han et al. (2016) found that average methylation rates of *RNF180* increased from normal mucosa, and atrophic gastritis to cancer of the stomach, while the conserve was seen for *RNF180* mRNA expression due to the infection of *Helicobacter pylori*. Among gastric samples, methylation of *RNF180* DNA promoter was negatively correlated with its expression (Han et al., 2016). Gradually, the expression level of *RNF180* decreased with increasing malignancy in gliomas (Cao et al., 2021). In this research, clinicopathological, and prognostic implications, and signal pathways of RNF180 expression were investigated, and the molecular mechanisms were clarified in EC.

## 2 Materials and methods

### 2.1 Cell culture and transfection

Esophageal squamous cancer cell line (KYSE-150) was purchased from Procell Company (Wuhan, China). It has been identified by short tandem repeats (STR) and proved to be free from *mycoplasma* pollution. Cells were cultured in RPMI 1640 growth medium supplemented with 10% fetal bovine serum (FBS, Cell-Box, Australia) in a humidified atmosphere of 5% CO_2_ at 37 °C. Cells were transfected with pcSLenti-EF1-EGFP-P2A-Puro-CMV-*RNF180*-3xFLAG-WPRE after seeding on dishes by Lipofectamine 3,000 (Thermo Fisher Scientific, United States). In order to check drug sensitivity, either TAXOL (a mitotic inhibitor, MedChemExpress, Cat. No. HY-B0015) or 5-fluorouracil (5-FU, thymidylate synthetase inhibitor, MCE, Cat. No. HY-90006) were used to treat cells. To investigate the effects of lipogenesis on chemosensitivity, we transfected pcDNA3.1-*ACC1*-3×Flag or pcDNA3.1-ACLY-3×Flag (HonorGene, China) into RNF180 transfectants.

### 2.2 Proliferation assay

A Cell Counting Kit-8 (CCK-8) kit (CT01B, Cellcook Biotech, Guangzhou, China) was employed to assess cell viability/cytotoxicity. Briefly, 3,500 cells/well were seeded into the 96-well plates, and cultured in medium for 0–48 h, then 10 μL of CCK-8 test kit were added for following 90-min incubation, and the absorbance was tested at 450 nm wavelength by a Multiskan™ FC Microplate Photometer (Thermo Fisher Scientific).

### 2.3 Apoptosis assay

We used 7-amino-actinomycin (7-AAD) combined with phycoerythrin (PE)-labeled Annexin V (559,763, BD Pharmingen, United States) to detect the externalization of phosphatidylserine by flow cytometry (Beckman Coulter, Brea, CA, United States). In brief, 1 × 10^5^ cells were collected, washed with PBS, and pelleted. PE-labelled Annexin V (5 μL, final concentration: 1 ug/mL) and 7-AAD (5 μL, final concentration: 50 μg/mL) were added to cell suspension, mixed, incubated for 15 min, and examined by flow cytometry. Finally, the results were analyzed by Flowjo V10 software.

### 2.4 Wound healing assay

8.5 × 10^5^ cells/well were seeded into a 6-well culture plate. When cells reached 80% confluence, surface was throughly scraped off in line with pipette tips. Subsequently, the cells were rinsed thrice with phosphate buffered saline (PBS) to ensure the complete removal of any broken cells. Finally, cells were cultured with FBS-free RPM1640. Cell migration photos were taken at the same location at 0 h and 24 h.

### 2.5 Transwell assay

Transwell chambers (3,422, Costar, Corning, United States) pre-coated with Matrigel^®^ Basement membrane matrix (356,234, Corning Life Sciences, MA, United States) were used for the cell invasion assay. 4.6 × 10^5^ cells were seeded in the upper layer of the chamber, and the medium containing 14% FBS was added as an inducer in the lower layer. After 48 h of incubation at 37°C, the chamber was washed and fixed with 4% paraformaldehyde (Beyotime, China). After crystal violet staining (Beyotime, China), cells were photographed under the microscope. For the migration experiment, except for adding Matrigel (Corning, United States) to the upper layer and incubating for 48 h, the steps are the same as those of the invasion experiment. ImageJ was used to analyze the data.

### 2.6 Nile red staining

4,500 cells were seeded on a glass slide, cultured for 12 h, washed with PBS three times, fixed with 4% paraformaldehyde, and dyed with Nile red dye (MCE, United States) for 15 min, and subsequently 4′, 6′-diamidino-2-phenylindole (DAPI, C1006, Beyotime, Shanghai, China) for 5 min, and sealed with anti-fade mounting medium (MCE, United States). The average fluorescence intensity of cells was analyzed by ImageJ.

### 2.7 Proteasome extract

Minute nuclear or cytosolic proteasome enrichment kit (Cat. No.PN-040, Invent Biotechnologies, Plymouth, MN, United States) was used to extract proteasomes. Briefly, we collected and resuspended 5 × 10^6^ cells in 450 µL buffer A. After that, we immediately transferred the cell suspension to the filter cartridge. And then, we inverted the sample for a few times and centrifuged at 16,000 g for 30 s. After the filter was removed, we vortexed briefly and centrifuged the collection tube at 16,000 g for 30 min. The supernatant (400 µL) was transferred into a fresh 1.5 mL microfungi tube with 400 µL buffer B added. The sample was vortexed for 120 s and followed by 10-min incubation on ice. After centrifugation at 10,000 g for 10 min, we completely removed supernatant, and added 50–150 µL of Minute™ Denaturing Protein Solubilization Reagent (WA-009) to resuspend the precipitate for the following Western blot.

### 2.8 Co-immunoprecipitation (Co-IP)

For Co-IP, cells were lysed in ice-cold RIPA reagent (Merck, United States) using a cell disruptor. Cell lysates (>1000 μg) were incubated with 500 μg the antibody against RNF180, ACC1 or ACLY, and then incubated with the Protein G agarose beads (37478S, Cell Signaling Technology, Shanghai, China). Beads were washed with RIPA reagent four times, collected, and mixed with the sample. After heating (95°C, 10 min), the protein samples were eluted and separated from the beads. The sample supernatant was used for following Western blot.

### 2.9 Subjects and pathology

All the tissues and plasma were obtained from the First Affiliated Hospital of Jinzhou Medical University. All tissues were preserved in 10% neutral formalin, embedded in paraffin, sectioned at 4 μm, and stained by hematoxylin and eosin (HE). Under the microscope, the representative parts of solid tumors were selected. Using tissue microarray (TMA), 2 mm tissue cores were punched out from each donor block and transferred to recipient blocks with 48 tissue cores (Azumaya kin-1, Tokyo, Japan). Tissue and cDNA microarrays of esophageal cancer and normal mucosa was also purchased from Shanghai Outdo Biotech (Shanghai) and used for immunohistochemistry and RT-PCR respectively. Informed consent was obtained from the patients in writing. No treatment was taken in these EC patients before operation. The Ethical Committee of the First Affiliated Hospital of Jinzhou Medical University approved our protocol.

### 2.10 Western blot

Cells and homogenized tissues were lysed in RIPA buffer and measured by Coomassie Brilliant Blue Protein Assay Kit (Biorad). After that, the protein was separated by electric field migration on SDS- polyacrylamide gel, and transferred to the Polyvinylidene fluoride (PVDF) membrane (Merck, United States). After being blocked by 4% milk for 1 h, it was incubated with the primary antibody (Supplementary Table S1) at 37°C for 2.5 h. The membrane was washed with Tris-Buffered Saline Tween-20 (TBST) for 13 min and incubated with the HRP (horseradish peroxidase)-labelled IgG antibody (Proteintech, United States) corresponding to the primary antibody. ECL detection reagent (Beyotime, China) was used to detect the membrane signal, and ImageJ was used to analyze the signal intensity. Here, GAPDH is glyceraldehyde 3-phosphate dehydrogenase in glycolysis and β-actin comprises along with microtubules, a major component of the cytoskeleton, both of which are regarded as internal control for cytosolic fraction. Lamin B is an important nuclear layer protein belonging to the nuclear fibronectin family, mainly involved in maintaining the structure and function of the nuclear membrane, which is considered as internal control for nuclear fraction. PSMC1 is a proteasome 26 S subunit, ATPase 1 and acts as an internal control for proteasomal fraction.

### 2.11 Immunohistochemistry (IHC)

Four-μm-thick slides of TMA were heated at 65°C, deparaffinized with xylene, rehydrated with alcohol, and then retrieved in target retrieval solution (Beyotime, China). After that, slides were incubated in methanol containing 2.5% hydrogen peroxide to block endogenous peroxidase activity, and to stop nonspecific binding by TBST containing 4.5% bovine serum albumin for 18 min. TMA slides were incubated with anti-rabbit RNF180 antibody (Genetix, United States), washed with TBST, and incubated with HRP-labelled IgG antibody (DAKO, United States) for 1.5 h, and the binding sites were visualized with diaminobenzidine (DAB, yellow color). Sections were dehydrated by ethanol, cleared by xylene, and mounted by neutral balsam after counterstaining with Mayer’s hematoxylin.

### 2.12 Enzyme-linked immunosorbent assay (ELISA)

We performed ELISA with RNF180 ELISA Kit (Jianglai, China) to determine the plasma RNF180 concentration. In short, 45 μL of standard and diluted serum samples were added to polystyrene microtiter plates containing anti-RNF180 antibody, and then 90 μL of detection antibody labeled with HRP was added to each well. After that, we discarded the liquid and wash the plate using washing buffer. Reaction substrate was dispensed into the plate and then stop buffer was added. The absorbance was detected at 450 nm wavelength.

### 2.13 Bioinformatics analysis

Expression data of EC patients were obtained from NCBI GEO database (GSE45670; platform: Affymetrix-GPL570) and analyzed in R studio 4.1.0 software. Expression, methylation, differential, related genes and prognostic significance of *RNF180* were analyzed with the xiantao platform (https://www.xiantaozi.com/), STRING (https://www. string-db. org/cgi/input?sessionId = bgxRenupC483&input_page_show_search = on), and UALCAN database (https://ualcan.path.uab.edu/analysis.html). The prognostic significance of *RNF180* was also explored by Kaplan-Meier plotter (https://kmplot.com/analysis/index. php?p = serviceandcancer = pancancer_rase). We constructed PPI network for differential genes and screened out the top ten hub genes. GO-KEGG and GSEA analysis were used to predict signal pathways.

### 2.14 Statistical criteria

We conducted three independent experiments in triplicate, and the data are expressed as mean ± standard deviation. The means were differentiated using Mann-Whitney U. Spearman correlation analysis was used for rank data. Survival curves generated by Kaplan-Meier plots was compared using log-rank statistics. The model of Cox’s proportional hazards was used to perform multivariate analysis. Statistics were considered significant at *p < 0.05*.

## 3 Results

### 3.1 Clinicopathological significances of *RNF180* mRNA expression in EC

**FIGURE 1:**
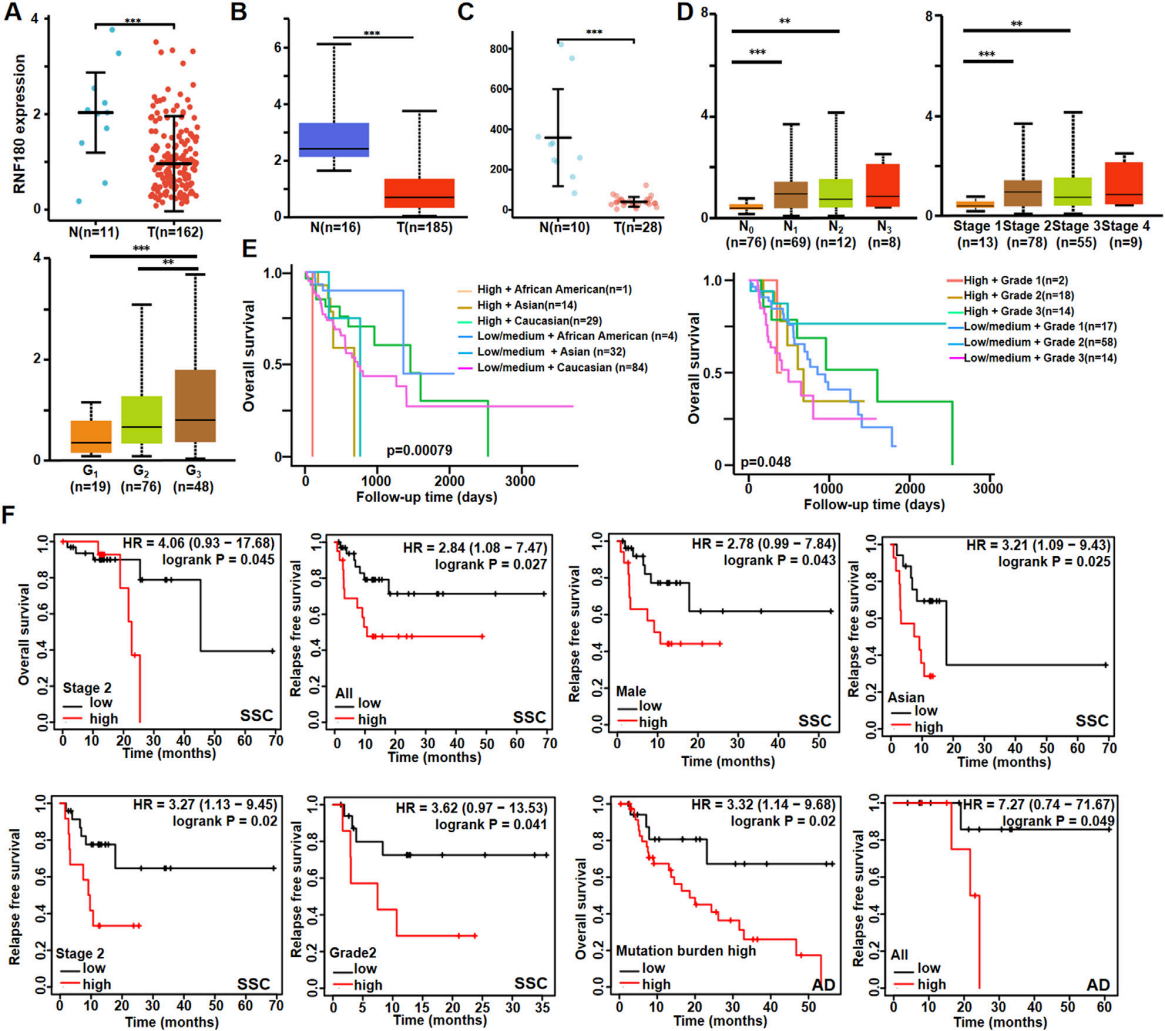
The clinicopathological significances of RNF180 mRNA expression according to bioinformatics analysis. The comparison of RNF180 mRNA expression was performed between esophageal normal and cancer tissues by xiantao **(A)**, UALCAN **(B)** and GEO **(C)** databases. It was also compared with clinicopathological features of esophageal cancer by UALCAN database **(D)**. The prognostic significance of RNF180 mRNA expression was explored by UALCAN database **(E)** and Kaplan-Meier plotter **(F)**. Note: N, normal; T, tumor; AD, adenocarcinoma; SCC, squamous cell carcinoma; *, p *< 0.05*; **, *p < 0.01*; ***, *p < 0.001*.

There was a lower *RNF180* mRNA expression in EC than in normal tissues according to xiantao (Figure 1A, *p < 0.05*), UALCAN (Figure 1B, *p < 0.05*) and GEO database (Figure 1C, *p < 0.05*). However, *RNF180* mRNA expression was higher in N_1_, N_2_ than in N_0_ patients, stage 2 and 3 than in stage 1 patients (Figure 1D, *p < 0.05*), and G_3_ than in G_2_ and G_1_ patients (Figure 1D, *p < 0.05*) (Figure 1D, *p < 0.05*) by UALCAN. Based on UALCAN database, *RNF180* mRNA expression was positively associated with a low overall survival rate, stratified by race and histological grading (Figure 1E, *p < 0.05*). Based on Kaplan-Meier plotter (Figure 1F), *RNF180* mRNA expression was positively linked to a low overall survival rate of the stage-2 SCC patients and AD patients with a mutation burden high (*p < 0.05*). It was the same for the relapse-free survival of all, male, Asian, stage 2, and grade 2 SCC patients and all AD patients (*p < 0.05*).

### 3.2 Clinicopathological significance of RNF180 methylation in EC

**FIGURE 2:**
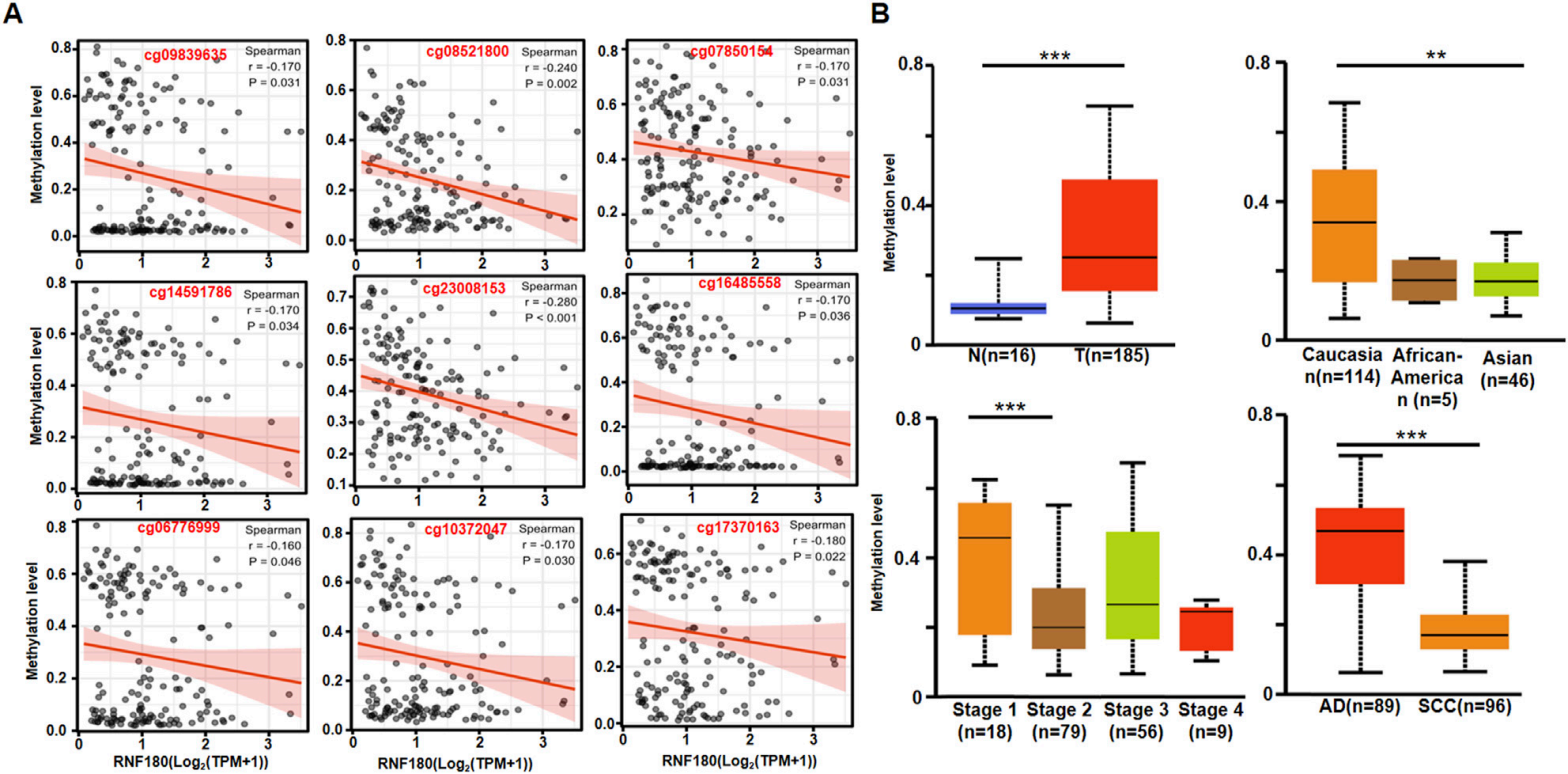
The clinicopathological significances of RNF180 methylation in esophageal cancer. The negative relationship between RNF180 mRNA expression and methylation was analyzed in esophageal cancer using the xiantao database (**A**, p < 0.05). Its methylation was also compared with clinicopathological features of esophageal cancer by UALCAN database (**B**, p < 0.05). Note: N, normal; T, tumor, AD; adenocarcinoma; SCC, squamous cell carcinoma; **, *p < 0.01*; ***, *p < 0.001*.

*RNF180* mRNA expression was negatively related to its promoter methylation at cg09839635, cg08521800, cg07850154, cg14591786, cg23008153, cg16485558, cg06776999, cg10372047 and cg17370,163 sites (Figure 2A, *p < 0.05*). *RNF180* methylation was higher in EC than in normal tissues (Figure 2B, *p < 0.05*), higher in Caucasians than Asians patients (Figure 2B, *p < 0.05*), higher in stage 1 than in stage 2 patients (Figure 2B, *p < 0.05*), and higher in AD than in SCC (Figure 2B, *p < 0.05*).

### 3.3 Genes and signal pathways associated with RNF180 in esophageal cancer

**FIGURE 3:**
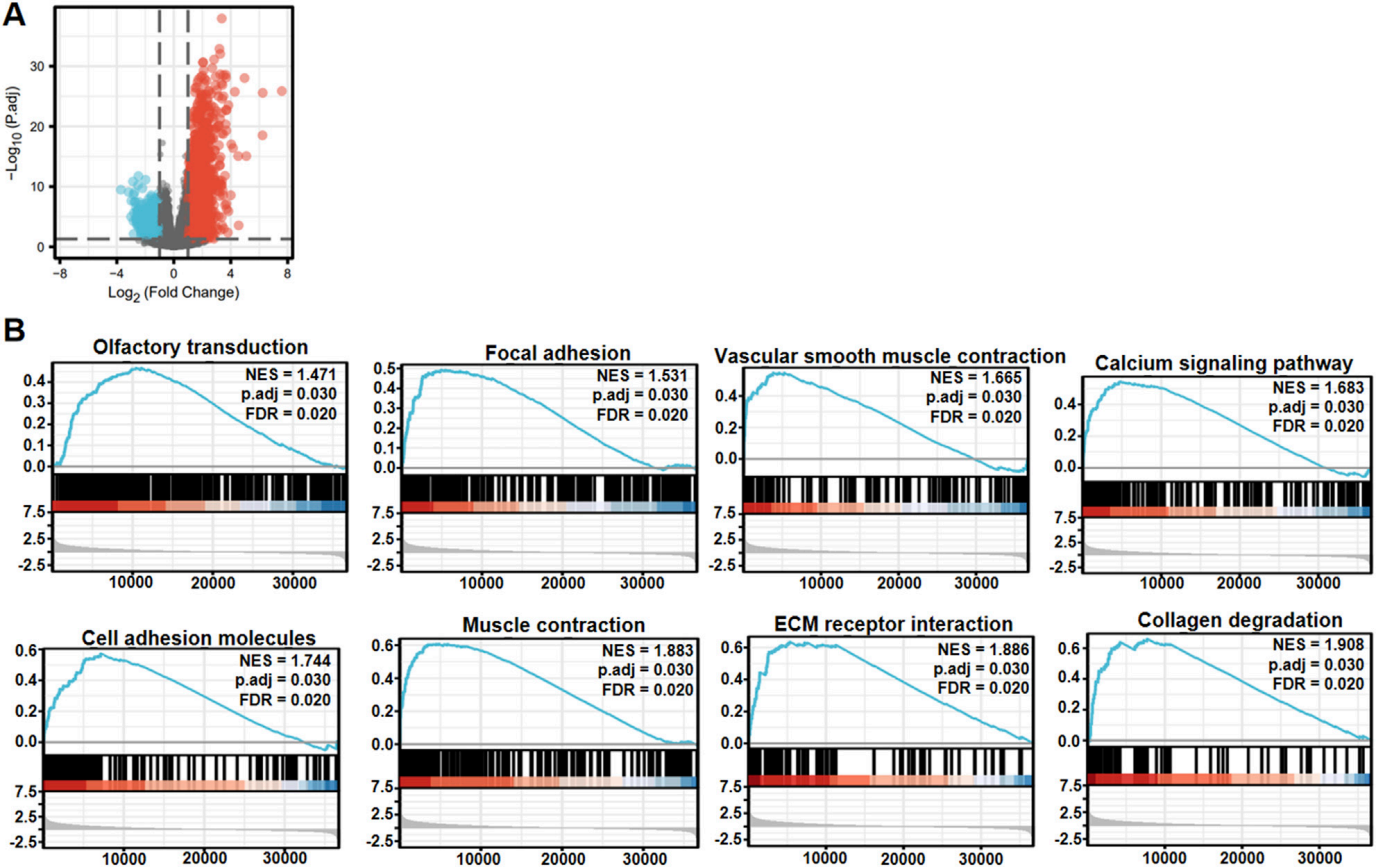
The differential genes and related signal pathways between low and high RNF180 expression in esophageal cancer. The volcano map of the differential genes was shown between low and high RNF180 expression in esophageal cancer **(A)**. These genes were subjected to the signal pathway analysis using GSEA **(B)**.

**FIGURE 4:**
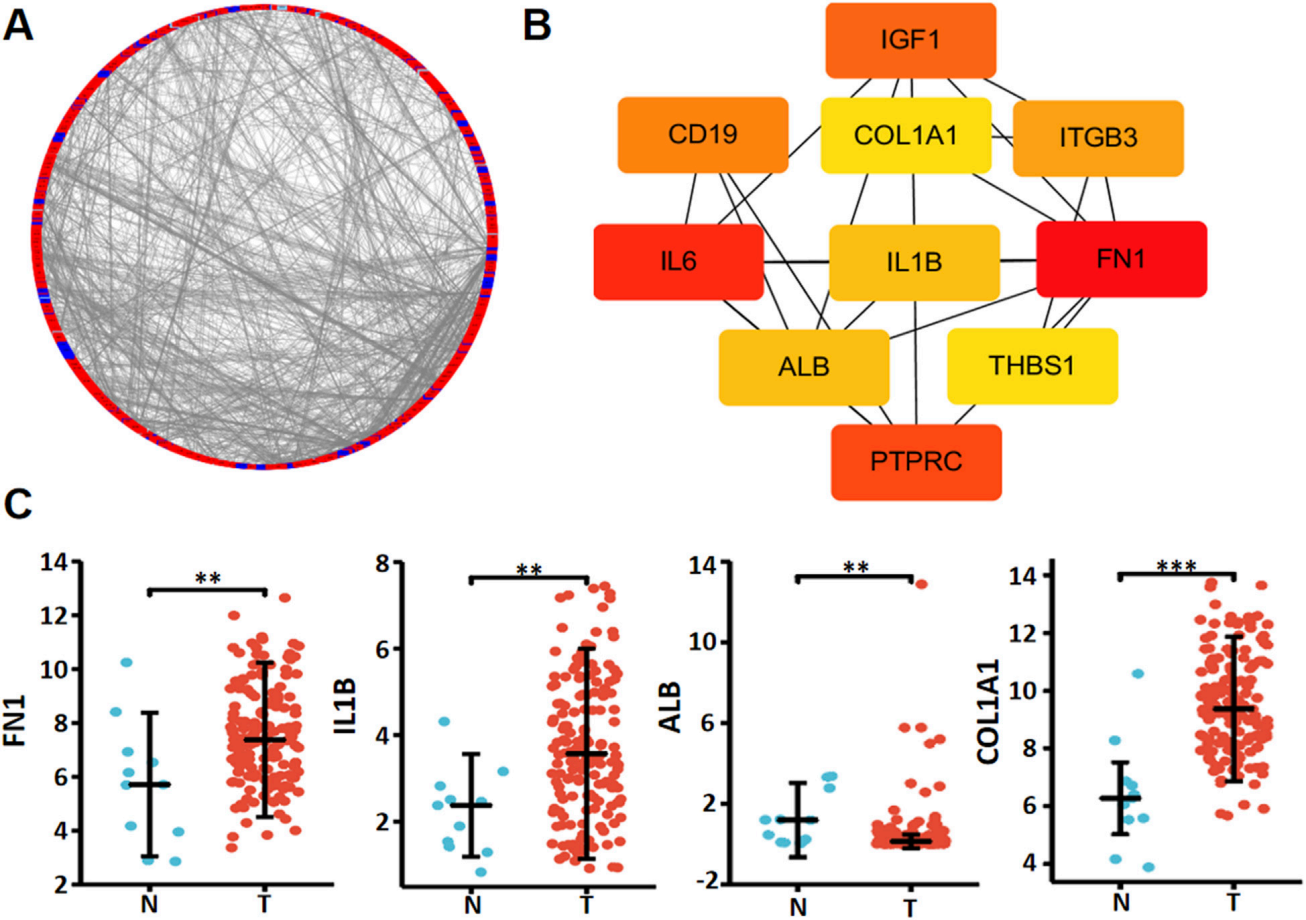
The hub genes of RNF180 in esophageal cancer. Both string and cytoscape were employed to screen the hub genes of RNF180 in esophageal cancer **(A)**. The hotspot hub genes were selected **(B)** and compared between esophageal cancer and normal tissues **(C)**. Note: N, normal; T, tumor; **, p < 0.01; ***, p<0.001.

Xiantao platform helped us find out which genes are differentially expressed between low and high levels of *RNF180* mRNA in EC and built a volcano map based on this information (Figure 3A). Based on GSEA analyses, top signal pathways included olfactory transduction, focal adhesion, vascular smooth muscle contraction, calcium signal pathway, cell adhesion molecules (CAMs), muscle contraction, extracellular matrix (ECM) receptor interaction and collagen degradation (Figure 3B, *p < 0.05*). PPI pairs were identified using STRING (Figure 4A), and the top 10 hub genes were identified using cytoscape (Figure 4B). As shown by xiantao, *FN1*, *IL1B*, and *COL1A1* were more expressed in EC than in normal samples (Figure 4C, *p < 0.05*). On the other hand, *ALB* showed the opposite pattern (Figure 4C, *p < 0.05*). FN1, IL1B, and COL1A1 contribute to the aggressiveness of EC by promoting invasion and metastasis (Fang et al., 2019; Ma et al., 2023; Chen et al., 2024). FN1 enhances cell adhesion, migration, and extracellular matrix (ECM) remodeling, facilitating tumor cell invasion (Ma et al., 2023). IL1B drives chronic inflammation, activates pro-tumorigenic signaling pathways (e.g., NF-κB), and induces epithelial-mesenchymal transition (EMT) (Chen et al., 2024). COL1A1 stiffens the tumor stroma, creating a pro-invasive microenvironment and supporting cancer cell proliferation and invasion (Fang et al., 2019). These molecules collectively exacerbate ECM dysregulation, inflammation, and tumor-stromal interactions, accelerating EC progression.

**FIGURE 5:**
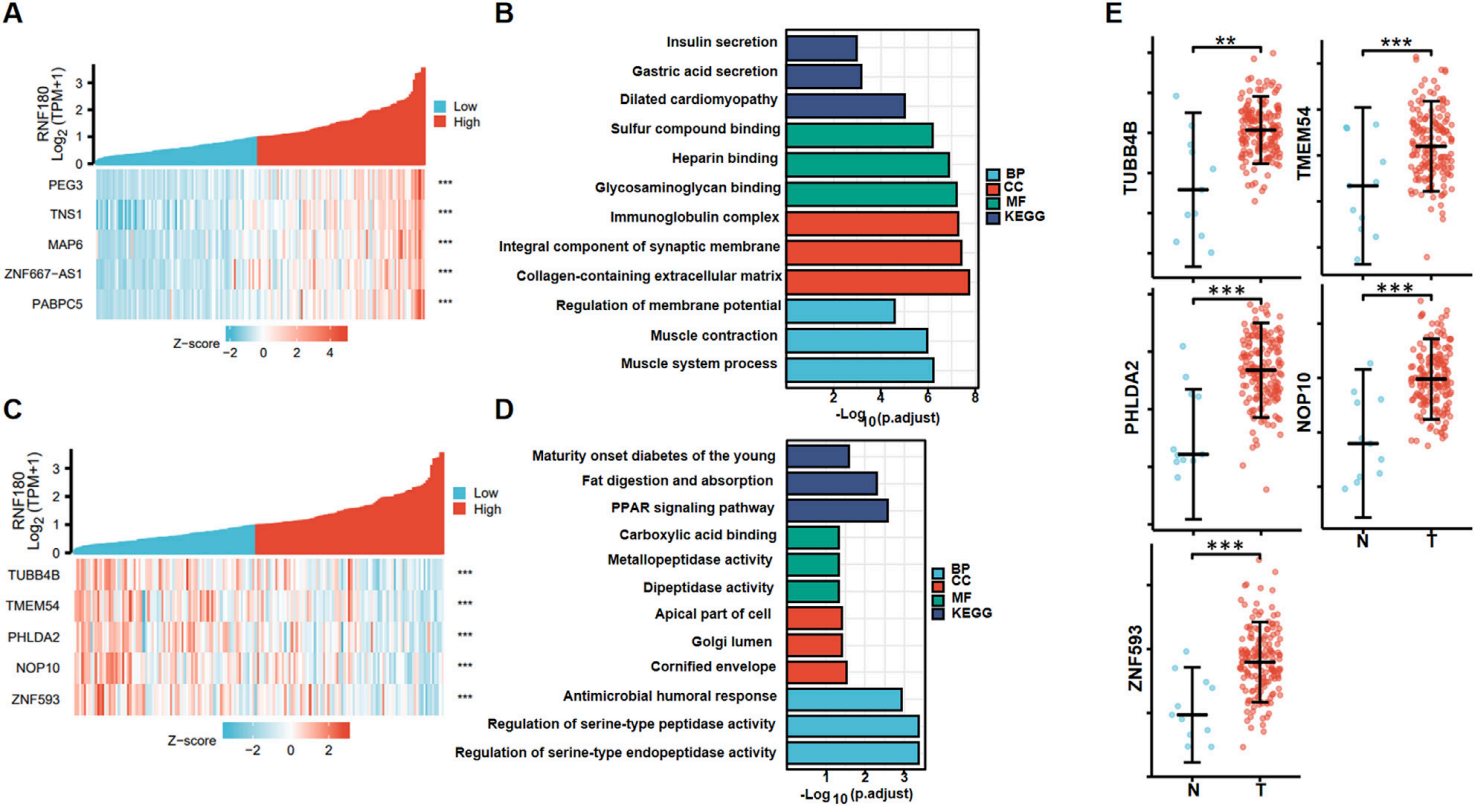
RNF180-related genes and signal pathways in esophageal cancer. The positively-related genes of RNF180 were screened **(A)**, and were classified into the signal pathway using xiantao database **(B)**. The negatively-related genes of RNF180 were screened **(C)**, and were classified into the signal pathway using xiantao database **(D)**. The expression of these genes was studied using xiantao platform **(E)**.

Figure 5A shows the top positively-correlated genes of *RNF180* in EC based on xiantao (*p < 0.05*). These genes were involved in gastric acid and insulin section, muscle and cardiomyopathy, glycoprotein binding, collagen and extracellular matrix (Figure 5B). Figure 5C shows the top negatively-correlated genes of *RNF180* in EC (*p < 0.05*). These genes were involved in fat digestion and diabetes, PPAR signal pathway, and dipeptidase activity (Figure 5D). All the top 5 *RNF180*-related genes (*TUBB4B*, *TMEM54*, *PHLDA2*, *NOP10*, and *ZNF593*) were more expressed in EC than in normal tissues (Figure 5E, *p < 0.05*).

### 3.4 *RNF180* expression in esophageal cancer and its clinical significances

**FIGURE 6:**
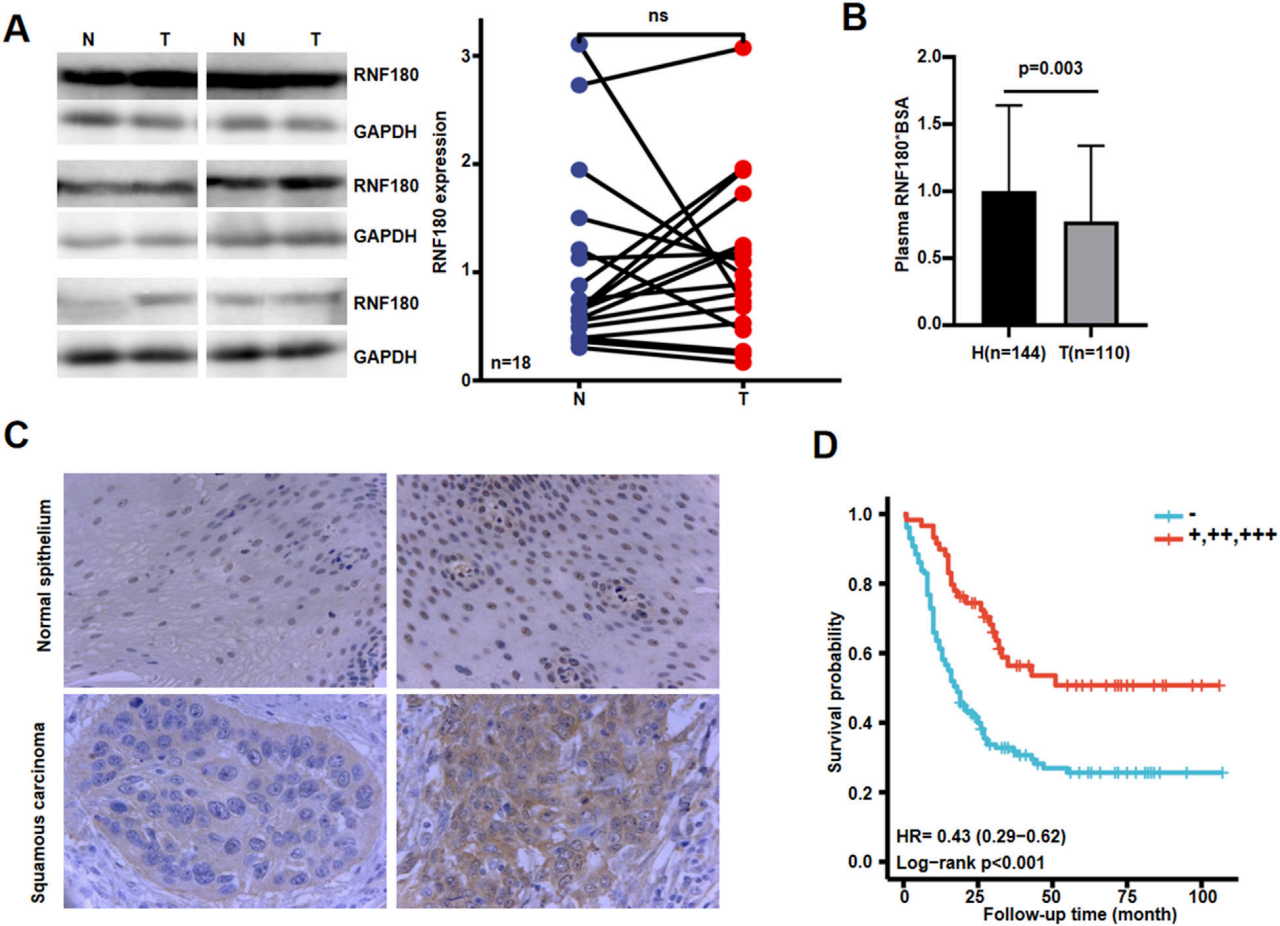
The clinicopathological significances of RNF180 protein expression in esophageal cancer. Western blot was used to detect RNF180 protein level in esophageal cancer **(A)**. Densimetric analysis showed no difference in RNF180 expression between esophageal cancer and normal tissues (**A**, *p > 0.05*). Plasma RNF180 was lower in healthy volunteer than esophageal cancer patients after the standardization by body surface area (**B**, *p < 0.05*). Immunohistochemically, RNF180 protein was positively expressed in esophageal squamous epithelial and cancer cells **(C)**. Kaplan-Meier curves and log-rank test were used to clarify the prognostic significance of RNF180 protein expression **(D)**. Note: N, normal; T, tumor; H, healthy volunteer; ns, not significant; BSA, body surface area; HR, hazard ratio.

Using Western blot, we found that RNF180 expression was similar in normal tissues and EC (Figures 6A, P *> 0.05*). Plasma RNF180 level was lower in EC patients than in healthy volunteers after the adjustment of body surface area (BSA) (Figure 6B, *p < 0.05*). RNF180 protein was positively expressed in either nuclei or cytosol of esophageal squamous epithelial cells and cancer cells by IHC (Figure 6C). The positive rate of RNF180 expression was 64.0% (215/336) in esophageal normal tissues and 58.4% (208/356) in EC with no statistical significance (Table 1, *p > 0.05*). There was a positive correlation between RNF180 expression and TNM stage, N stage, and T stage of EC (Table 2, *p < 0.05*). On univariate analysis, sex, T stage, N stage, TNM stage, and *RNF180* expression were positively correlated with unfavorable overall survival of EC patients (Figure 6D; Table 3, *p < 0.05*). In multivariate analysis, the expression of *RNF180*, TNM stage, and sex were found to be independent factors in EC patients (Table 3, *p < 0.05*).

**TABLE 1:** RNF180 expression during esophageal carcinogenesis.

Groups	n	RNF180 expression				
		-	+	++	+++	PR (%)
Normal tissue	336	121	154	49	12	64.0
Esophageal cancer	356	148	144	56	8	58.4

Note: PR, positive rate.

**TABLE 2:** The relationship between RNF180 protein expression and clinicopathological characteristics of esophageal cancer.

Clinicopathological features	n	RNF180 expression				PR (%)	p-value
		-	+	++	+++		
Sex							0.938
Female	53	24	17	8	4	54.7	
Male	303	124	127	48	4	59.1	
Age (years)							0.966
<65	201	82	85	30	4	59.2	
≥65	153	65	58	26	4	57.5	
Histological grade							0.838
Ⅰ	69	29	25	13	2	58.0	
Ⅱ-Ⅲ	251	95	109	41	6	62.2	
T stage							**0.005**
T1	23	14	6	3	0	39.1	
T2	60	29	21	8	2	51.7	
T3	253	102	108	39	4	59.7	
T4	13	1	6	4	2	92.3	
N stage							**0.009**
N0	153	78	52	21	2	49.0	
N1	109	36	51	19	3	67.0	
N2	73	26	31	14	2	64.4	
N3	16	6	7	2	1	62.5	
TNM stage							**0.001**
I	24	18	4	2	0	25.0	
II	138	62	54	20	2	55.1	
III	178	62	78	32	6	65.2	
IV	8	2	5	1	0	75.0	

Note: PR, positive rate. Boldface indicates *p* < 0.05, which are statistically significant.

**TABLE 3:** The survival analysis for the esophageal cancer patients.

Clinicopathological features	Univariate analysis			Multivariate analysis		
	β	HR (95% CI)	p-value	β	HR (95% CI)	p-value
Sex (male vs female)	0.598	1.819 (1.245–2.656)	**0.002**	0.687	1.988 (1.113–3.549)	**0.020**
Age (≥65 vs <65 years)	−0.17	0.874 (0.642–1.117)	0.24	−0.11	0.895 (0.605–1.326)	0.581
T stage (T1-2 vs T3-4)	0.715	2.044 (1.339–3.120)	**0.001**	−0.04	0.962 (0.525–1.764)	0.900
N stage (N0-1 vs N2-3)	0.807	2.241 (1.643–3.057)	**<0.001**	0.428	1.534 (0.836–2.815)	0.167
Histological grade (I-II vs III)	0.018	1.019 (0.738–1.406)	0.911	0.214	1.238 (0.803–1.911)	0.334
TNM stage (I-II vs III-IV)	0.784	2.190 (1.635–2.934)	**<0.001**	0.629	1.876 (1.015–3.465)	**0.045**
RNF180 expression (-vs +, ++, +++)	0.861	2.365 (1.517–3.686)	**<0.001**	0.726	2.067 (1.285–3.326)	**0.003**

Note: HR, hazard ratio; CI, confidence interval. Boldface indicates p < 0.05, which are statistically significant.

### 3.5 Biological phenotypes and relevant molecules of EC cells were affected by RNF180 expression in EC cells

**FIGURE 7:**
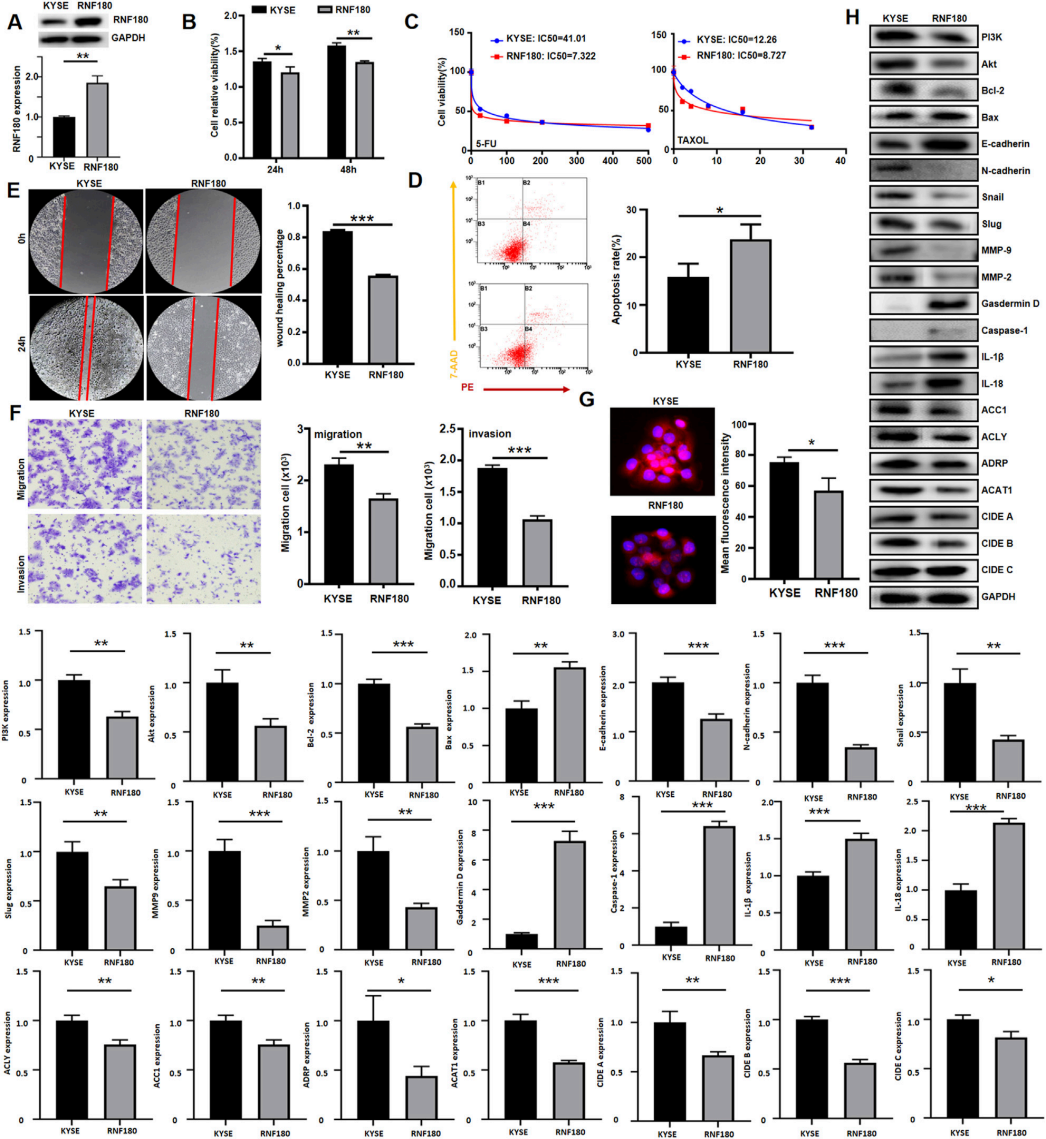
The effects of RNF180 expression on the aggressive phenotypes and phenotype-related protein of esophageal cancer cells. After transfection of RNF180-expressing plasmid, RNF180 expression became stronger than the control in KYSE-150 cells by Western blot **(A)**. The transfectants were subjected to the function assays of proliferation, chemosensitivity to 5-FU and TAXOL, apoptosis, migration, invasion, and lipid droplet formation by CCK-8 **(B, C)**, Annexin V/7-AAD staining **(D)**, wound healing **(E)**, transwell chamber **(F)**, and Nile red staining **(G)** respectively (*p < 0.05*). The phenotype’s proteins were screened by Western blot **(H)**. Note: KYSE, KYSE-150; *, *p < 0.05*; **, *p < 0.01*; ***, *p < 0.001*.

After transfected with *RNF180*-expressing plasmid, KYSE-150 had the overexpression of RNF180 protein, evidenced by Western blot (Figure 7A). RNF180-overexpressing cells showed decreased proliferative capacity, compared to parental cells (Figure 7B, *p < 0.05*). Overexpression of RNF180 increased the chemosensitivity to 5-FU and TAXOL (Figure 7C). After ectopic RNF180 overexpression, high levels of apoptosis were observed in KYSE-150 cells (Figure 7D, *p < 0.05*). In wound healing (Figure 7E, *p < 0.05*) and transwell assays (Figure 7F, *p < 0.05*), RNF180 overexpression decreased the migration and invasion capacity of KYSE-150 cell. RNF180 expression caused less lipid droplets formation than parental cells by Nile red staining (Figure 7G, *p < 0.05*). In Figure 7H, we see that RNF180 overexpression decreased the expression of PI3K, Akt, Bcl-2, N-cadherin, Snail, Slug, MMP-2, MMP-9, ACC1, ACLY, ADRP, ACAT1, CIDEB, CIDEA, but increased the expression of Bax, Caspase-1, Gasdermin D, IL-18, IL-1β and E-cadherin.

**FIGURE 8:**
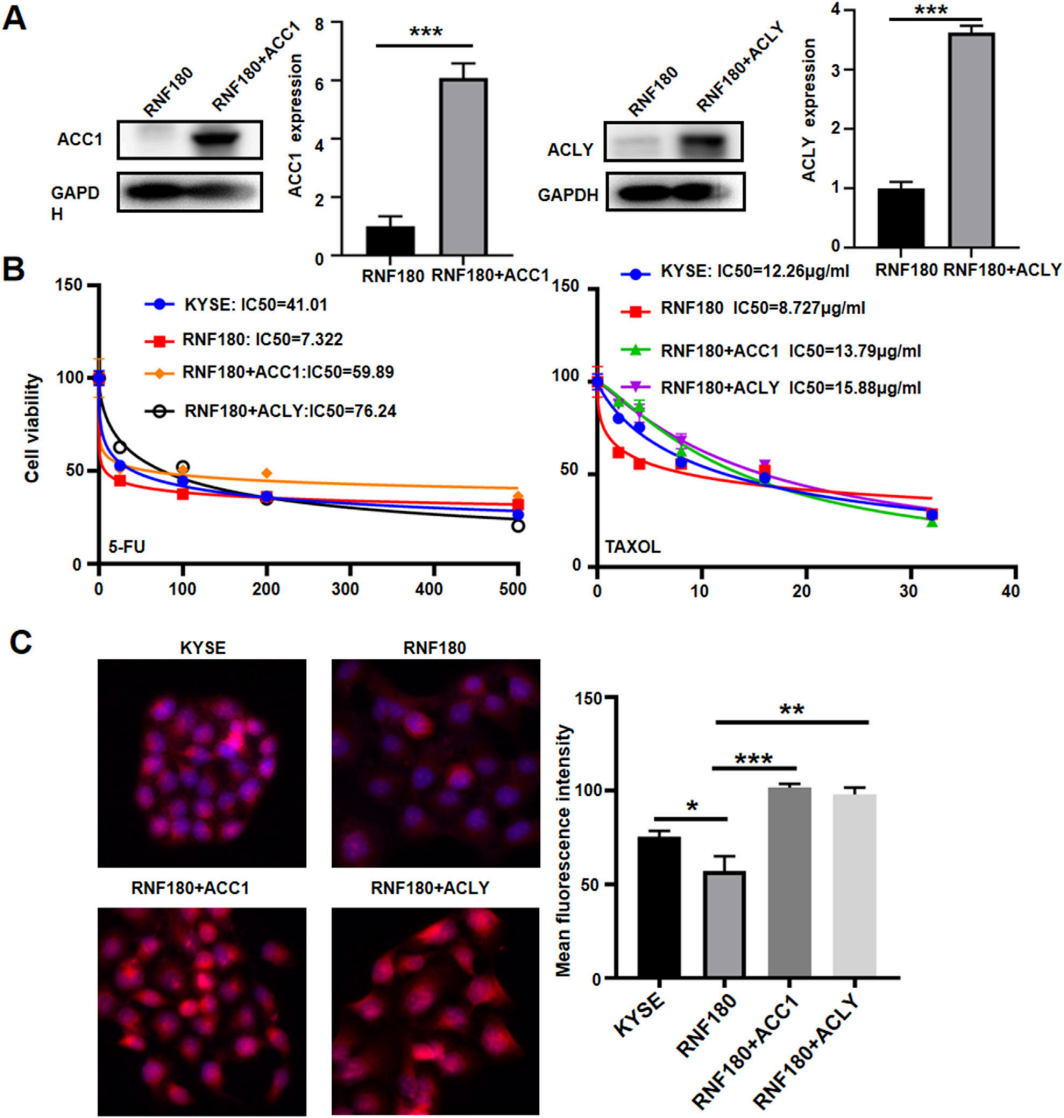
The effects of ACC1 and ACLY on the RNF180-mediated chemosensitivity and lipid droplet formation. ACC1 was overexpressed in ACC1 transfectants and ACLY was overexpressed in ACLY transfectants of KYSE-150 cells, evidenced by Western blot **(A)**. After the treatment of 5-FU and TAXOL, KYSE-150 cells and transfectants were subjected to CCK-8 **(B)** and Nile red staining **(C)** respectively. Note: KYSE, KYSE-150; *, *p < 0.05*; **, *p < 0.01*; ***, *p < 0.001*.

In order to confirm the impact of ACC1 and ACLY on RNF180-induced drug resistance to chemotherapy and lipid droplets formation, we increased their expression levels in KYSE-150 cells, evidenced by Western blot (Figure 8A). According to CCK-8 (Figure 8B, *p < 0.05*) and Nile red staining (Figure 8C, *p < 0.05*), either ACC1 or ACLY increased the chemoresistance against 5-FU and TAXOL as well as lipid droplets formation in KYSE-150 cells.

### 3.6 proteasomal degradation of ACLY and ACC1 proteins was mediated by RNF180 in EC cells

**FIGURE 9:**
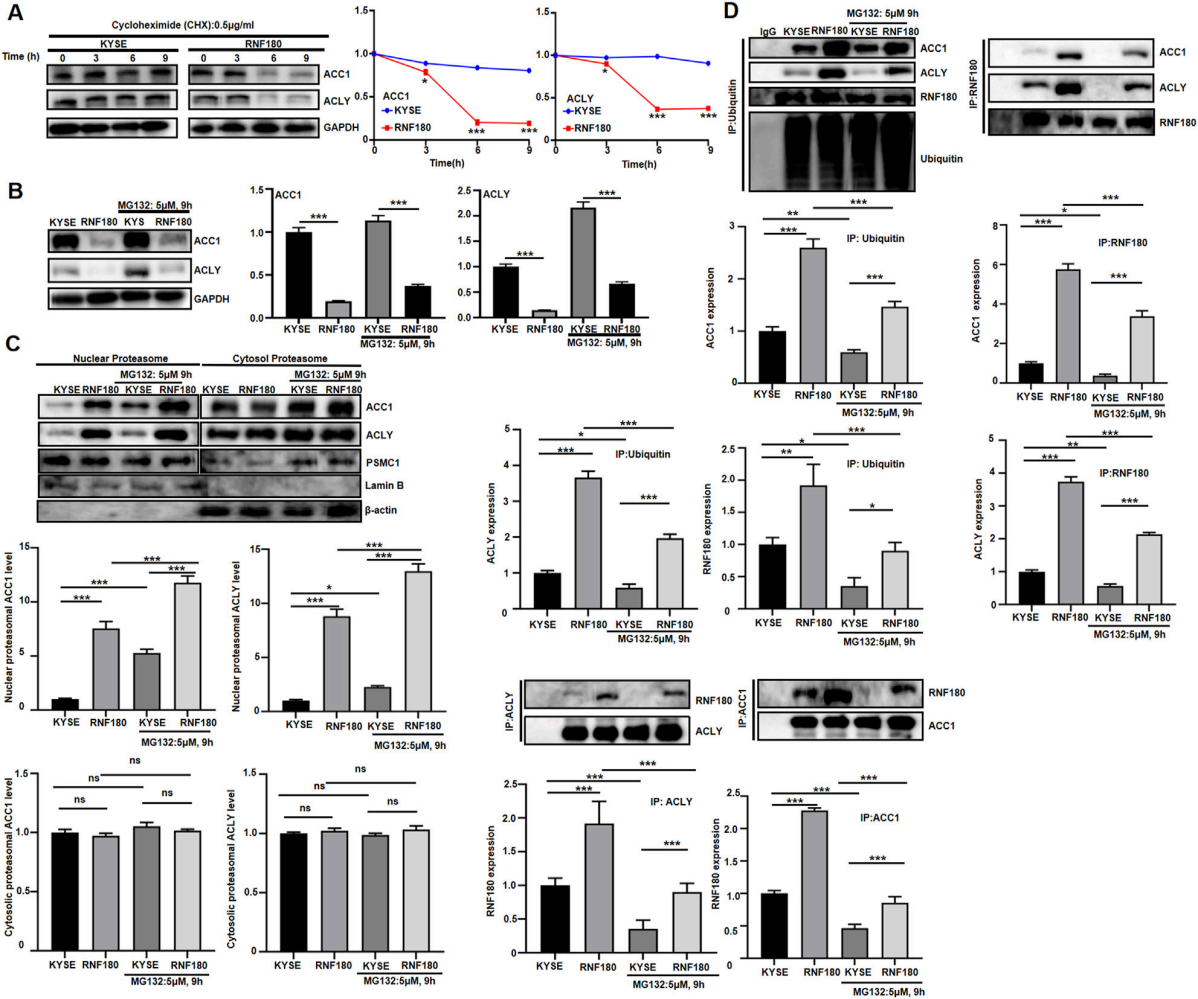
RNF180 destablized ACLY and ACC1 proteins via proteasomal degradation. KYSE cells and RNF180 transfectants were treated with cycloheximide (CHX, 0.5 μg/ml, **A**) or MG132 (5 μM, 9h, **B**), and followed by Western blot. KYSE cells and RNF180 transfectants were subjected to proteasomal extract and subsequent Western blot, even treated with MG132 **(C)**. After co- immunoprecipitation, Western blot was performed in KYSE cells and KYSE transfectants, even treated with MG132 **(D)**. Note: KYSE, KYSE-150; PSMC1, a marker for proteasome; Lamin B, a marker for nuclear fraction; GAPDH and β-actin, a marker for cytosolic fraction; IP, immunoprecipitation; ns, not significant; *, *p < 0.05*; **, *p < 0.01*; ***, *p < 0.001*.

After cycloheximide treatment, ACLY and ACC1 expression were low in *RNF180* transfectants, compared to KYSE-150 cells (Figure 9A, *p < 0.05*). MG132 strengthened the expression of ACC1 and ACLY although their expressions were lower in KYSE-150 cells than in RNF180 transfectants (Figure 9B, p < 0.05). RNF180 transfectants had higher levels of ACC1 and ACLY protein in their nuclear proteasomes than KYSE-150 cells, which was strengthened by MG132 treatment (Figure 9C, *p < 0.05*). In cytosol proteasome, the expressions of ACC1 and ACLY were similar between RNF180 transfectants and their parental cells regardless of the treatment with MG132 (Figure 9C, *p > 0.05*). Ubiquitylated ACC1 and ACLY appeared more abundant in RNF180 transfectants than in KYSE-150 cells although MG132 reduced the levels in KYSE-150 cells and RNF180 transfectants according to Co-IP (Figure 9D, *p < 0.05*). ACC1 and ACLY bound more to RNF180 in RNF180 transfectants than KYSE-150 cells although MG132 reduced the levels in KYSE-150 cells and RNF180 transfectants according to Co-IP (Figure 9D, *p < 0.05*).

## 4 Discussion and conclusion

RNF180 is a RING finger membrane-bound E3 ubiquitin ligase, whose tail anchored to endoplasmic reticulum (Ogawa et al., 2008). With the help of the E2 ubiquitin-conjugating enzyme UBE2E1, RNF180 ubiquitinates ZIC2 and leads to its degradation by proteasomes in hepatocellular carcinoma cells (Sun W. et al., 2021). Han et al. (2016) reported that *RNF180* mRNA level were significantly lower in gastric cancer than in non-tumor tissues and that it was lower in hypermethylated than hypomethylated samples. *RNF180* hypermethylation was found to involve in hepatocellular carcinogenesis using a genome-wide DNA methylation approach (Zhang et al., 2014). A higher hypermethylation of *RNF180* was detectable in the plasma DNA of gastric cancer patients than in healthy volunteers as well (Zhang et al., 2014). Dai et al. (2025) demonstrated that circulating methylated RNF180 and SFRP2 could serve as diagnostic biomarkers for gastric cancer by the random forest model, in line with the report of plasma SHOX2, SEPTIN9, RNF180, and EPO methylation about EC (Liu et al., 2024). Liu et al. (2020) showed that *RNF180* expression loss was positively correlated with its promoter methylation, T stage, and adverse prognosis of non-small cell lung cancer patients as an independent predictor. In line with *RNF180* hypo-expression in lung (Liu et al., 2020), colorectal (Wei et al., 2021) and ovarian (Zhao et al., 2024) cancers, and low plasma level of RNF180 in EC, we found that *RNF180* mRNA expression was lower in esophageal cancer than normal tissues, opposite to *RNF180* methylation level. Plasma RNF180 might derive from protein shuttling between the plasma membrane (Zheng and Jiang, 2022) and nucleus or cell death (necrosis, apoptosis, pyroptosis and so forth). According to our data, RNF180 might be localized to the nuclear and cytosolic proteasomes. In our immunostaining, its nuclear distribution was principally observed in normal esophageal epithelial cells, but its cytosolic immunoreactivity was observed in cancer cells, illustrating that its nucleocytosolic translocation might play an important role in esophageal carcinogenesis despite no difference in RNF180 protein expression between esophageal cancer and normal tissues. However, *RNF180* mRNA was positively associated with N stage, pathological stage, histological grading, or *RNF180* promoter methylation in esophageal cancer. There was a positive correlation between RNF180 protein expression and T, N and TNM stage of EC. In EC, *RNF180* methylation was inversely related to TNM stage and adenocarcinoma subtype. Taken together, in esophageal cancer, downregulated expression of RNF180 contributed to tumor development and progression, possibly due to its promoter methylation. *RNF180* expression and methylation level were employed to indicate the aggressive behaviors of EC. However, its positive correlation with aggressive characteristics might be attributable to its reactive feedback overexpression in advanced cancers.

Deng et al. (2014) reported that *RNF180* promoter hypermethylation was significantly related to lymph node metastasis and postoperative overall survival of gastric cancer patients, in line with the plasma finding (Nie et al., 2023). Deng et al. (2016a) demonstrated that among 400 patients with gastric cancer, four hypermethylated CpG sites (−116, −80, +97, and +102) of *RNF180* were significantly associated with survival. Xie et al. (2015) found that the patients with seven or fewer hypermethylated CpG sites of *RNF180* promoter had higher survival rates. In our research, we found that regardless of race, histological subtype, or grade, *RNF180* mRNA expression was negatively associated with overall and relapse-free survival. In contrast, a positive correlation was found between RNF180 protein and overall survival in EC patients. In combination with these data, we believe that RNF180 may be used to predict the prognosis of the patients with EC in clinical practice. However, the paradoxical data about the prognostic significances of RNF180 mRNA and protein might be due to the better therapeutic efficacy for RNF180-protein-positive EC patients because RNF180 was found to promote chemosensitivity of EC cells in our study.

Sun L. et al. (2021) identified that DNMT3A was ubiquitinated by RNF180 and then degraded by the proteasome in gastric cancer cells, finally to reduce viability and motility. Wu et al. (2020) found that overexpression of RNF180 inhibited STAT3 phosphorylation in gastric cancer cells by ubiquitination and destroying RhoC. Wei et al. (2021) showed that WISP1 was ubiquitinated and degraded by RNF180 expression in colorectal cancer, inhibiting proliferation and promoting apoptosis. Deng et al. (2016b) reported that methylation of *RNF180* DNA promoters might dramatically influence such malignant biological characteristics of gastric cancer cells as proliferation, invasion, anti-apoptosis and tumorigenicity, in agreement with our data. Cheung et al. (2012) revealed that re-expression of RNF180 suppressed cell growth and induced apoptosis through upregulation of antiproliferation regulators MTSS1 and CDKN2A, as well as proapoptotic mediator TIMP3. In the current study, enhanced expression of RNF180 suppressed the proliferation, resistance to chemotherapeutic agents, migration and invasion, and induced apoptosis in EC cells. Consequently, we speculated that RNF180 might someday serve as a therapeutic target of EC.

The expression of RNF180 can be detected in several adult tissues, as well as in the lens and brain of immature animals (Ogawa et al., 2008). Kabayama et al. (2013) showed that due to Rines’ interaction with monoamine oxidase A (MAO-A) and promotion of its degradation, RNF180 regulated brain MAO-A subset, monoamine levels, and emotional behavior. Zagajewska et al. (2018) performed genome-wide association studies (GWAS) and found that *RNF180* rs72769818 SNP had a protective effect for Pseudoexfoliation syndrome because ubiquitination and proteasomal degradation were involved in the control of neuritogenesis. Here, we found that *RNF180* was involved in olfactory transduction, focal adhesion, vascular smooth muscle contraction, calcium signal pathway, cell adhesion molecules, ECM receptor interaction, gastric acid and insulin section, glycoprotein binding, collagen and extracellular matrix, PPAR signal pathway, peptidase activity, which might be due to the ubiquitination degradation of key enzymes or signal proteins during these biological processes and account for the regulatory effects of RNF180 on the aggressive phenotypes of EC cells.

PI3K/Akt pathway is commonly over-activated in cancers, and participates in cell proliferation and anti-apoptosis (He et al., 2022; Sanaei et al., 2022). By interacting with Bax on mitochondrial membrane, Bcl-2 suppresses Bax-mediated activation of mitochondrial voltage-dependent anion channels (Zheng, 2017). Overexpression of RNF180 inhibited proliferation in EC cells and increased apoptosis by decreasing Bcl-2/Bax and inactivating PI3K/Akt. As previously reported, pyroptosis is characterized by Caspase-1-mediated cell death and resemble those of inflammatory programmed necrosis with Gasdermin D and IL-18 overexpression (Arakelian et al., 2022). It has been found that Twist promotes epithelial-mesenchymal transition (EMT) by overexpressing E-cadherin and underexpressing N-cadherin (Fedele et al., 2022). Matrix metalloproteinases (MMPs) took responsibility for the degradation of the extracellular matrix components (Zheng et al., 2006). Consequently, RNF180 might promote Caspase-1-dependent pyroptosis, and suppress EMT of EC cells by increasing slug and snail expression. MMP-2 and MMP-9 protein hypoexpression may also explain the inhibitory impacts of RNF180 on invasion and metastasis of EC cells. In colorectal cancer cells, LPCAT2-mediated lipid droplet formation caused the chemoresistance, which was also assisted by prothymosin α (Cotte et al., 2018; Jin et al., 2021). Metastasis-associated in colon cancer 1 resulted in the chemoresistance of gastric cancer cells to oxaliplatin by up-regulating fatty acid synthase expression (Duan et al., 2017). Chemoresistance is closely linked to the enzymes involved in *de novo* fatty acid synthesis, including ACC1 or ACLY (Sur et al., 2019). Several factors mediate the assembly of lipid droplets in the liver and peritoneum, including ACAT1, ADRP, and CIDEs (Fan et al., 2020; Kasano-Camones et al., 2020; Ayyagari et al., 2022). In the study, we found that RNF180 promoted chemosensitivity of EC cells. Previously, lipid droplet formation facilitated the chemoresistance in EC cells (Yun et al., 2023; Yun et al., 2024). Therefore, we performed Nile staining and Western blot for the key enzymes of lipogenesis, such as ACC1 and ACLY. In EC cells, RNF180-mediated lipid droplet formation was likely associated with ADRP, CIDEA, and CIDEB expression. Lipogenesis induced by RNF180 might be linked to ACC1 and ACLY expression. In combination of the increase in lipid droplet formation and chemoresistance in RNF180 transfectants, which was induced by ACC1 and ACLY overexpression, we speculated that RNF180 might suppressed the drug resistance of EC cells by inhibiting lipogenesis. Consistent with our findings, Wang et al. (2021) also found that RNF180 overexpression conferred cisplatin sensitivity in gastric cancer cells. As a result of these discoveries, we hypothesized that RNF180 weakened both lipogenesis and lipid droplet assembly, therefore contributing to chemosensitivity.

In gastric cancer cells, RNF180 ubiquitinated DNA methyltransferase 1, and 3α for proteasome-mediated degradation to inhibit viability, motility or metastasis, suppressed STAT3 phosphorylation by ubiquitination and proteasomal degradation of RhoC, and strengthened the malignancy suppression and ferroptosis facilitation of BCL6 (Wu et al., 2020; Sun et al., 2021b; Guo et al., 2023; Zhang et al., 2023). In colorectal cancer cells, Wei et al. (2021) showed that WISP1 was ubiquitinated and degraded by RNF180 expression to inhibit proliferation and increase apoptosis. Zhao et al. (2023) found that RNF180 inhibited the proliferation, anti-apoptosis, and EMT of osteosarcoma cells by inducing chromobox homolog 4 (CBX4) ubiquitination, which downregulated Kruppel-like factor 6 and upregulated RUNX family transcription factor 2. Ding et al. (2022) found that RNF180 inhibited cell proliferation, tumor growth, and energy metabolism by degrading c-myc in a ubiquitin-dependent manner in non-small cell lung cancer cells. Wang et al. (2024) reported that RNF180 degraded ALKBH5 *via* ubiquitination and ALKBH5 facilitated SMARCA5 hypoexpression via m6A modification, finally to aggravate colon inflammation and Th17/Treg imbalance in ulcerative colitis. Zhao et al. (2023) found that RNF180 inhibited the nuclear translocation of SOX2 by promoting ubiquitination of IPO4 to impair IPO4/SOX2 complex stability and inhibit SOX2-mediated aggressiveness of ovarian cancer. Here, we demonstrated that RNF180 might decrease the stability of ACC1 and ACLY proteins, which was blocked by MG132, a proteasomal inhibitor. Additionally, ACC1 and ACLY existed in proteasomes with high ubiquitination and could interact with RNF180 proteins. Taken together, we speculated that RNF180 facilitated the proteasomal degradation of ACC1 and ACLY, which subsequently suppressed lipogenesis and subsequent chemoresistance of EC cells.

Although the clinicopathological significances of RNF180 expression and its effects on aggressive phenotypes have been explored, the following scientific issues should be resolved in the future work: (1) There was no difference in RNF180 protein expression between esophageal cancer and normal tissues, but its mRNA expression was downregulated in EC; (2) Demethylation agents should be used to verify whether methylation can influence the transcription of RNF180 in EC cells; (3) It should be investigated which kinds of cells plasma RNF180 come from and why its plasma level is low in EC. (4) The partner proteins of RNF180 should be clarified for the proteasomal degradation of ACLY and ACC1. (5) The synergically effects of the proteasomal (MG132), lipid droplet formation (triacsin C), ACLY and ACC1 inhibitors with 5-FU or cisplatin should be observed in xenograft tumor model of EC cells in nude mice. These above-mentioned points are the limitation of the present study.

In conclusion, RNF180 expression was downregulated in EC, possibly due to its promoter methylation, and closely linked to the aggressively pathological behaviors and prognosis of EC. In esophageal cancer cells, RNF180 is believed to suppress proliferation, migration, invasion, and EMT and induce apoptosis and pyroptosis. ACC1- and ACLY-mediated lipogenesis and lipid droplet assembly might be inhibited by RNF180 in esophageal cancer cells, finally to result in chemosensitivity.

## Data Availability

The original contributions presented in the study are included in the article/Supplementary Material, further inquiries can be directed to the corresponding author.
