# Supplementary material for: RNF180 weakened the lipid droplet formation and subsequent chemoresistance by destabilizing ACC1 and ACLY in esophageal cancer
Source: Front Pharmacol. 2025 Apr 22;16:1525431. doi: 10.3389/fphar.2025.1525431 (PMC12052773; doi:10.3389/fphar.2025.1525431)
Supplement: Supplementary file 1 [file Table1.docx]

Supplementary Material

**Supplementary table 1** The primary antibodies used in this study

| **Name** | **Dilution** | **Source** | **Company** | **Catalog number** |
| --- | --- | --- | --- | --- |
| GAPDH | 1:2000 for WB | Mouse | Proteintech, USA | 60004-1-Ig |
| RNF180 | 1:1000 for WB; 1:100 for IHC; 5ug for co-IP | Rabbit | Genetex, USA | GTX04092 |
| PI3K | 1:1000 for WB | Rabbit | Proteintech, USA | 20584-1-AP |
| Akt | 1:1000 for WB | Rabbit | Proteintech, USA | 10176-2-AP |
| Bcl-2 | 1:1000 for WB | Rabbit | Proteintech, USA | 26593-1-AP |
| Bax | 1:2000 for WB | Rabbit | Proteintech, USA | 50599-2-Ig |
| E-cadherin | 1:20000 for WB | Rabbit | Proteintech, USA | 20874-1-AP |
| N-cadherin | 1:2000 for WB | Rabbit | Proteintech, USA | 22018-1-AP |
| Snail | 1:1000 for WB | Rabbit | Proteintech, USA | 13099-1-AP |
| Slug | 1:5000 for WB | Rabbit | Proteintech, USA | 12129-1-AP |
| MMP-9 | 1:1000 for WB | Rabbit | Proteintech, USA | 10375-2-AP |
| MMP-2 | 1:1000 for WB | Rabbit | Proteintech, USA | 10373-2-AP |
| Gasdermin D | 1:2000 for WB | Rabbit | Proteintech, USA | 20770-1-AP |
| Caspase-1 | 1:2000 for WB | Rabbit | Proteintech, USA | 22915-1-AP |
| IL-1β | 1:2000 for WB | Rabbit | Proteintech, USA | 16806-1-AP |
| IL-18 | 1:2000 for WB | Rabbit | Proteintech, USA | 10663-1-AP |
| ACLY | 1:2000 for WB; 10 ug per co-IP | Rabbit | Proteintech, USA | 15421-1-AP |
| ACC1 | 1:2000 for WB; 10 ug per co-IP | Rabbit | Proteintech, USA | 21923-1-AP |
| ACAT1 | 1:2000 for WB | Rabbit | Proteintech, USA | 16215-1-AP |
| ADRP | 1:2000 for WB | Rabbit | Proteintech, USA | 15294-1-AP |
| CIDE A | 1:1000 for WB | Rabbit | Proteintech, USA | 13170-1-AP |
| CIDE B | 1:2000 for WB | Rabbit | Proteintech, USA | 27600-1-AP |
| CIDE C | 1:2000 for WB | Rabbit | Proteintech, USA | 12287-1-AP |
| PSMC1 | 1:500 For WB | Rabbit | Proteintech, USA | 11196-1-AP |
| Ubiquitin | 1:1000 for WB; 10 ug per co-IP | Rabbit | Proteintech, USA | 10201-2-AP |
| β-actin | 1:1000 for WB | Rabbit | Proteintech, USA | 20536-1-AP |
| Lamin B | 1:5000 for WB | Rabbit | Proteintech, USA | 12987-1-AP |

*Note*: WB, western blot; IHC, immunohistochemistry; co-IP, co- immunoprecipitation.
